# Supplementary material for: Vital Needs of Dutch Homeless Service Users: Responsiveness of Local Services in the Light of Health Equity
Source: Int J Environ Res Public Health. 2023 Jan 31;20(3):2546. doi: 10.3390/ijerph20032546 (PMC9915996; doi:10.3390/ijerph20032546)
Supplement: Supplementary file 1 [file ijerph-20-02546-s001.zip › ijerph-2156842_S1_Supplementary_Tables.pdf]

Related article:
Van Everdingen, C.; Peerenboom, P.B.; Van der Velden, K.; Delespaul, PAEG.
Vital Needs of Dutch Homeless Service Users: Responsiveness of Local Services in the Light of Health Equity.
Int. J. Environ. Res. Public Health **2023**, *20*, 2546. https://doi.org/10.3390/10.3390/ijerph20032546

Abbreviations:
ASS: Algorithmic Summary Scales; BI: Basic Interviews; CIA: Clinical Integrating Assessments.
CAN: Camberwell Assessment of Needs. Recoded results: N: no need; M: met needs; U: unmet needs.
CMH: InterRAI Community Mental Health questionnaire.
ETHOS: European Typology of Homelessness and Housing Exclusion.
HOP-TR study: Homeless People Treatment and Recovery Study.
HSup: Homelessness Supplement; HSupN: newly defined HSup assessments for this paper.
MH: mental health; MI: mental illness; SMI: severe mental illness.
SCIL: Screener for Cognition Intelligence and Learning disabilities.

Domain	Variable	Source		Operationalization
Homelessness history	Homelessness typology	ETHOS	ASS	*Roofless:* rough sleepers and night shelter users. *Houseless:* in temporary homelessness accomodation, in long-term homelessness support, or living independently with ambulant support.
	Homelessness duration	HSup	BI	Original HSup variable to assess the duration of current episode of homelessness/houselessness.
	Previous homelessness	HSup	BI	Original HSup variable to assess the presence of previous episodes of homelessness/houselessness
Demographics	Age	CMH	BI	Original CMH variable
	Gender	CMH	BI	Original CMH variable
	Migration background	CMH	BI	Original CMH variable
Education	Education level	HSup	BI	Categorization of 'Highest education achievements' in three levels conform to Netherlands Statistics. *Low* indicates that education was limited to 3 years of secondary school or vocational training (lowest level). *Middle* indicates completion of the last 2 or 3 years of secondary school or vocational training (middle level). *High* points to completion of vocational training (highest level) or a university degree.
Work	Work status	HSup	BI	Reflects the current work status and orders the working experience of subjects without work: Never employed; persistently unemployed; currently unemployed; employed with benefit; (regularly) employed; retired; student.
Income	Income status	HSup	ASS	Recoded HSup variable, based on 'income source' to convey procedural information about obtaining benefits: no benefit; benefit inquiry submitted; benefit inquiry granted.
	Trade-offs	CMH	BI	Original CMH variable, to assess trade-offs because of essential needs in the last 30 days.

Table S1: Background characteristics.

Domain	Variable	Source		Operationalization
Basic needs	Home	CIA	CIA	Rights-based custom assessing the optimal residence after leaving the homeless services.
	Paid work	HSup	BI	The Dutch ministry of Education defined the need of medium or high educational achievements for sustainable chances of a qualified regular job in the Dutch labor market. Based on that norm, the highest education level was used for computing the need of work support.
	Administration support	SCIL	BI	Needs reflecting the functional illiteracy screening: the ability to handle written information in Dutch.
	Concurrent basic needs	HSup	ASS	Sum of 4 no/yes assessments of the need of a home, income, low-literacy support, and work support. The need of a home was stated at 100% since all subjects met the ETHOS criteria. Financial problems and income uncertainties were common. Therefore, the need of an income was also stated at 100%.
Health needs	Physical monitoring needs	CMH	ASS	Sum of 11 no/yes variables to describe care needs due to chronic physical health conditions (cardiovascular, diabetes, gastrointestinal, hard drug use in last year, infectious, malignancy, neurological, respiratory, sex work, thyroid, under-/overweight).<br>Migraine, musculoskeletal problems, and cured malignancies were not included.<br>Figure 2a presents the means of the sum scores, table 2A presents the dichotomized results.
	Mental health vulnerabilities	CIA	ASS	Sum of 10 no/yes variables to assess the burden of MH problems inducing MH care needs (anxiety, addiction, intellectual impairments, trauma, depression, psychosis, agitation, problematic personality, neurocognitive impairments, somatization).<br>Figure 2a presents the means of the sum scores, table 2A presents the dichotomized results.
	Mental illness	CIA	CIA	Any transdiagnostic mental health features, except intellectual impairments or addiction. (Anxiety, trauma, depression, psychosis, agitation, problematic personality, neurocognitive impairments, somatization)
	Substance use	CIA	CIA	Transdiagnostic features of 'Addiction'.
	Intellectual impairments	CIA	CIA	Transdiagnostic features of 'Intellectual impairments'.
	Trauma impact	CMH	ASS	Sum of the dichotomized CMH variables to assess the impact of trauma in daily life<br>(4 items: intense fear, fear of family member, unexplained injuries, safety concerns).
	Self-harm indicators	CMH	ASS	Sum of the dichotomized CMH variables to assess violent behavior towards oneself<br>(2 items: self-harm thoughts, self-harm attempts).
	Expressions of violence	CMH	ASS	Sum of the dichotomized CMH variables to assess expressions of violent behavior towards others<br>(3 items: violence threatening, violent behavior, violence ideation).
Concurrent needs	Concurrent basic needs	HSup	ASS	Categorical variable, based on the results of two no/yes variables describing the presence of any basic needs in addition to the need of an income and a home.<br>Value labels: no additional needs; only paid work; only administration; paid work & administration.
	Concurrent health needs	CMH, CIA	ASS	Categorical variable, based on the results of two no/yes variables describing the presence of any mental and physical health needs.<br>Value labels: no health needs; only physical; only mental; physical & mental.
Divergence	Single needs	ASS	ASS	No/yes variable, representing the results of four no/yes variables assessing the presence of any physical, mental, administration, and paid work needs respectively. 'Single needs' reflects that the presence of needs is limited to one domain, while needs in the other three domains were absent.
	Double, triple, quadruple needs	ASS	ASS	Similar. 'Double needs' reflects that needs are present in two domains, while needs in the other two domains were absent. Etc.

Table S2: Basic and health needs

Domain	Variable	Source		Operationalization
Social services	Paid work support	CMH HSup	ASS	The CMH variable 'work arrangement' assessed the presence of a working arrangement: absent; present, aimed at regular work; present, aimed at supported work. We recoded the basic interview data to count the presence of working arrangements aimed at regular work.
	Substitute decision maker	CMH	BI	Original CMH variable.
	Administrative support	CMH HSup	ASS	Sum of two no/yes variables to count the presence of social service contacts offering administrative support or a substitute decision maker.
General health services	Any physician visits	CMH	BI	Any physician visits in the last 90 days.
Mental health services	MH service engagement	HSupN	CIA	The engagement of MH services in the past 6 months was assessed, based on relevant basic interview data. Value labels: no engagement; monodisciplinary; multidisciplinary.
	Latest contact in last year	CMH	BI	Based on original CMH variable, assessing the time since the latest ambulant MH service contact. Looks back to the last month and the last year.
	Latest contact in last week	CMH	ASS	The Basic Interviews collected data about the character and time since the deliverance of various MH treatment modalities (7 items, such as individual therapy, group therapy, alcohol & drugs treatment). The presence of any MH service contacts in the last week reflects the sum of the 7 no/yes variables.
	Intended MH treatment	HSupN	ASS	Sum of two no/yes variables counting the presence of consistent care guidance and/or intended MH therapies scheduled to start in the next month.

Table S3: Service presence and deliverables

Domain	Source		Operationalization
Paid work	CMH<br>HSup	ASS	We used work status and presence/focus of working arrangements for computing the responsiveness to support needs aimed at a regular job.<br>N was scored if the work status revealed that the subject was retired or student.<br>M was scored if the work status revealed that the subject had a regular job. M was also scored if the work status revealed that the subject was persistently or currently unemployed, had social service support and a working arrangement aimed at regular work.<br>U was scored if the subject was unemployed, and the support aimed at supported work. U was scored in the cases that were left.
Administration	CMH<br>HSup	ASS	We computed the presence of administrative support. The need of low-literacy support was based on the functional literacy screening results.<br>N was scored if functional illiteracy was absent.<br>M was scored if any administrative support and functional illiteracy were present.<br>U was scored in the absence of any administrative support, while functional illiteracy was present.
Physical health	CMH<br>HSup	ASS	We computed the presence of any physician visits in the last 90 days and considered the results in relation to the presence of physical needs.<br>N was scored if physical care needs were absent.<br>M was scored if physical care needs were present and there was at least one physician visit in the last 90 days.<br>U was scored if physical needs were present, but physician visits were absent.
Mental health	CMH<br>HSup	ASS	Previous research resulted in the assessment MH-related care needs in the HOP-TR sample: *Absent* uncovered needs*, Conditional* care needs in relation to Mental Illness*,* and long-term, *Intensive,* adaptive care needs in relation to Severe Mental Illness. In the basic interviews, data was collected about the latest ambulant MH service contacts: no contact in the last year; 31 days ago or more; 30 days or less.<br>We computed the presence of ambulant MH services in relation to the MH-related care needs:<br>N was scored if MH-related care needs were absent.<br>In the presence of conditional needs, M was scored if there was any ambulant MH contact in the last year.<br>In the presence of intensive needs, M was scored if a multidisciplinary team was engaged and there was any ambulant MH contact in the last week.<br>U was scored in the remaining cases.

Table S4: Service responsiveness to vital needs
